# A Demographic Survey of Pertrochanteric Fractures Based on the Revised Arbeitsgemeinschaft für Osteosynthesefragen/Orthopedic Trauma Association (AO/OTA) Classification Using 3D CT Scan Images

**DOI:** 10.7759/cureus.33572

**Published:** 2023-01-09

**Authors:** Mitsuaki Noda, Shunsuke Takahara, Ryota Nishida, Keisuke Oe, Atsuyuki Inui, Shin Osawa, Takehiko Matsushita

**Affiliations:** 1 Department of Orthopedics, Nishi Hospital, Kobe, JPN; 2 Department of Orthopedics, Hyogo Prefectural Kakogawa Medical Center, Kakogawa, JPN; 3 Department of Orthopedics, Kobe University Graduate School of Medicine, Kobe, JPN; 4 Department of Orthopedics, Nobuhara Hospital, Tatsuno, JPN

**Keywords:** computed tomography, ao/ota classification, survey, plain radiograph, pertrochanteric fracture

## Abstract

Introduction

A demographic survey of femoral pertrochanteric fractures provides several important information for the healthcare system of a country since this fracture is commonly seen in the elderly and has a poor postoperative functional prognosis that is a burden on society. The importance of accurately classifying pertrochanteric fractures as stable or unstable cannot be understated. However, the use of plain radiograph images alone is known to underestimate fracture severity with low inter- or intra-observer agreement. Computed tomography (CT) images offer information for a more accurate classification of pertrochanteric fractures. With this three-dimensional (3D) CT-based study using the revised Arbeitsgemeinschaft für Osteosynthesefragen/Orthopedic Trauma Association (AO/OTA) classiﬁcation, the purpose of this study is to elucidate the epidemiological demography of patients with pertrochanteric fractures.

Material and methods

We retrospectively collected 808 patients from five hospitals, classified into two groups: stable (A1) or unstable (A2). Age, gender, fracture laterality, and surgery timing were identified as epidemiological variables. Patients with both preoperative plain radiographs and 3D CT scans were included in the study. The exclusion criteria were AO/OTA A3 type fractures, pathological fractures, previous ipsilateral surgery, 60 years old or younger, and conservatively treated patients. The primary outcome involved detailing the total number of fractures based on classification (A1 or A2) and variables. The secondary outcome involved a comparison between the A1 and A2 groups.

Results

The mean age of patients at the time of surgery was 85 years (range: 61-103 years). There were 637 female and 171 male patients. There were 463 left-sided fractures and 345 right-sided fractures. Of the 808 patients, 371 (45.9%) were classified to have A1 fractures, and 437 (54.1%) had A2 fractures. The age at surgery, gender, fracture laterality, and surgery timing between the A1 and A2 groups were compared. The mean and standard deviation of the age at surgery for patients in the A1 and A2 groups were 84.9±7.7 and 86.9±6.8, respectively. The number of patients for each age distribution of 61-69, 70-74, 75-79, 80-84, 85-89, 90-94, and 95 or older for the A1 and A2 groups was 18 and 7, 18 and 12, 43 and 44, 76 and 82, 107 and 132, 79 and 110, and 30 and 50, respectively, showing that the difference in categorial distribution was statistically significant (p=0.002). Overall, 278 females and 93 males were classified to have A1 fractures compared with 359 females and 78 males with A2 fractures (p=0.01). There were 166 right-sided and 205 left-sided stable A1 fractures and 179 right-sided and 258 left-sided A2 fractures (not significant (NS)). Among the total number of A1 and A2 surgeries by month, the most were in December with 77 surgeries (37 and 40, respectively), and the least was in June with 37 (18 and 19, respectively). The seasonal classification for A1 and A2 surgeries is as follows: spring with 172 (74 and 98, respectively), summer with 150 (70 and 80, respectively), autumn with 193 (90 and 103, respectively), and winter with 208 (97 and 111, respectively) (NS).

Conclusion

In this demographic study of 808 patients with pertrochanteric fractures classified by 3D CT images, 371 had A1 fractures and 437 had A2 fractures. A2 fractures were significantly more in females with an age peak of 85-89 years.

## Introduction

A demographic survey of femoral pertrochanteric fractures provides several important information for the healthcare system of a country. First, pertrochanteric fractures rank as one of the most common surgically treated injuries in the elderly population, considering that the incidence of hip fractures is projected to reach six million globally by 2050 [1]. Second, the poor functional prognosis related to pertrochanteric fractures has been a burden on society, where 20%-30% of patients die within a year postoperatively and 50%-60% suffer from disability. Only 30%-40% of patients fully recover to their previous functional level [2]. There is a growing negative impact of pertrochanteric fractures and their consequences on the field of medicine, society, and the economy.

The importance of accurately classifying pertrochanteric fractures as stable or unstable cannot be understated as postoperative outcomes, such as fracture nonunion, failure, or hemoglobin levels, are different [3-5]. The revised Arbeitsgemeinschaft für Osteosynthesefragen/Orthopedic Trauma Association (AO/OTA) classiﬁcation (A1: stable, A2: unstable) is one of the most widely used classification systems [6]. Most studies involving the classification of pertrochanteric fracture have used plain radiograph images [7-9]. However, the use of plain radiograph images alone may underestimate fracture severity because of the difficulty in accurately assessing the complex morphology of the posterior femoral intertrochanteric region, particularly on the sagittal view. Furthermore, interobserver reliability in such studies remains “moderate to fair” [7,8].

We believe that accurate classification of pertrochanteric fractures is important in projecting the future burden on the individual patient and to society as well, as long as the majority of epidemiological studies classify the fractures into stable or unstable groups. Computed tomography (CT) images provide clearer and more detailed information for a more accurate classification of pertrochanteric fractures when compared to plain radiographs alone [10,11]. With the recent advancements in technology, three-dimensional (3D) CT images have become possible [12]. With our 3D CT-based revised AO/OTA classification study, we aim to correctly elucidate the epidemiological demography of 808 patients with pertrochanteric fractures.

## Materials and methods

Data source and collection

Data were collected retrospectively from orthopedic patients of five hospitals (Nishi Hospital, Kakogawa Medical Center, Kasai Municipal Hospital, Suzuran Hospital, and Nakatani Orthopedic Hospital). A consecutive series of 989 patients with femoral pertrochanteric fractures recruited in the Surgical Entry Database in these five hospitals from January 2015 to December 2020 were extracted using the keyword “pertrochanteric fracture.” Only patients with preoperative two-directional plain radiographs (anteroposterior and lateral views) and CT scans (3D CT) were included in the study.

The exclusion criteria were as follows: AO/OTA A3 type fractures, pathological fractures due to neoplasm or infection, presence of ipsilateral surgical treatment, 60 years old or younger at the time of surgery, and conservatively treated patients.

Data for analysis

**Figure 1:**
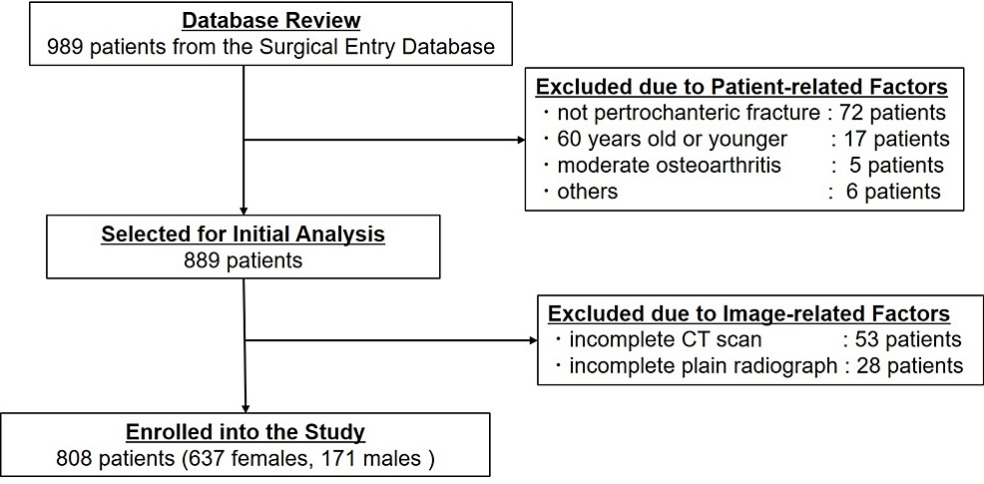
CONSORT flowchart of patient selection showing the inclusion and exclusion process CONSORT: Consolidated Standards of Reporting Trials, CT: computed tomography

Out of 989 patients identified from the Surgical Entry Database, a total of 808 patients who met the inclusion criteria for analysis were enrolled in the study. One hundred were excluded due to patient-related factors, namely, not pertrochanteric fractures (72 patients), 60 years old or younger (17 patients), presence of moderate osteoarthritic changes (five patients), and other reasons such as the presence of hereditary deformities (six patients). Another 81 patients were excluded due to the absence of a complete set of images (CT set: 53 patients, plain radiographs: 28 patients) (Figure 1).

Data classification

Based on the revised AO/OTA classiﬁcation [6], patients with pertrochanteric fractures were classified into two groups: stable (A1) or unstable (A2). A surgeon experienced with the revised AO/OTA classiﬁcation examined both CT and plain radiograph images.

Variables

Epidemiological data (age, gender, fracture laterality, and surgery timing) were identified as variables in the two fracture groups. Regarding age, patients were grouped into seven chronological age categories: 61-69, 70-74, 75-79, 80-84, 85-89, 90-94, and 95 or older [13]. Gender and fracture laterality were also determined. In addition, the distribution was surveyed by month and season, based on the timing of the surgical intervention.

Primary and secondary outcomes

The primary outcome involved detailing the total number of fractures based on the classification (A1: stable, A2: unstable), age categories, gender, laterality of fracture, and date of surgery in months (from January to December) and the following four seasons: spring (March-May), summer (June-August), autumn (September-November), and winter (December-February). The secondary outcome involved a comparison of the abovementioned variables between the A1 and A2 groups.

Statistical analysis

The Mann-Whitney U test was used to compare the age characteristics of patients between the stable (A1) and unstable (A2) groups. Gender and laterality of the fracture site were analyzed using the chi-squared test. The monthly and seasonal variables in the two groups were reported as frequencies and compared using the Student’s t-test with normality confirmed using the Kolmogorov-Smirnov test. A p value of <0.05 was considered statistically significant.

Statistical analysis was performed using EZR (Saitama Medical Center, Jichi Medical University, Saitama, Japan), a graphical user interface for R (The R Foundation for Statistical Computing, Vienna, Austria).

Ethics, funding, and potential conflicts of interest

This multicenter study was approved by the Research Ethics Board of each of the five hospitals mentioned above and was conducted in accordance with the 1964 Helsinki Declaration, with informed consent waived (represented by the ethical committee with approval number 2021-1). There are no competing interests to declare. No external funding was received for this study.

## Results

The mean age of the 808 patients at the time of surgery was 85 years (range: 61-103 years). The patients were grouped into seven age categories, namely, 61-69, 70-74, 75-79, 80-84, 85-89, 90-94, and 95 or older, and the number of patients for each age category was 25, 30, 87, 158, 239, 189, and 80. The 85-89 age group had the largest number of patients with 239. There were 3.7 times more female patients than male patients (637 females to 171 males). There were 463 left-sided fractures and 345 right-sided fractures (Table 1).

**Table 1:** Patient demographics and comparison of variables between A1 and A2 fractures ‡: p values for the A1 and A2 groups analyzed by gender and laterality of fracture site †: Mean age and standard deviation *: p value of age groups

Variable	A1 fracture	A2 fracture	Total	P value
Gender (number)	-	-	-	p=0.01‡
Female	278	359	637	-
Male	93	78	171	-
Age group† (years)	84.9±7.7	86.9±6.8	-	p=0.002*
61-69	18	7	25	-
70-74	18	12	30	-
75-79	43	44	87	-
80-84	76	82	158	-
85-89	107	132	239	-
90-94	79	110	189	-
95 and above	30	50	80	-
Laterality (number)	-	-	-	p=0.28‡
Right	166	179	345	-
Left	205	258	463	-

Comparison of variables between A1 and A2 fractures

**Figure 2:**
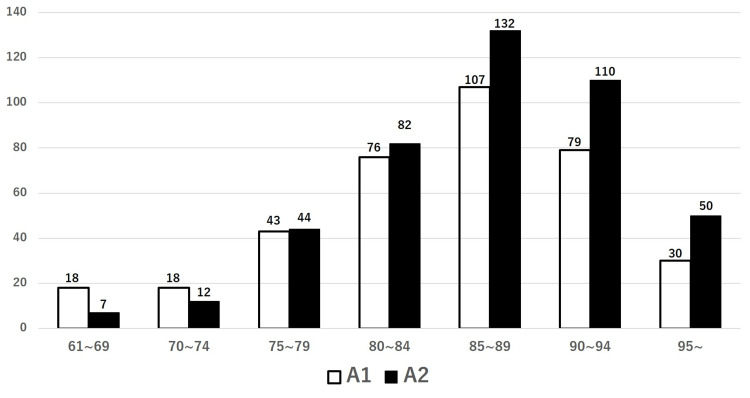
Number of patients classified as A1 or A2 per age group The number of fractures increases with age and is more pronounced in the A2 group compared with the A1 group. A1: stable, A2: unstable

**Figure 3:**
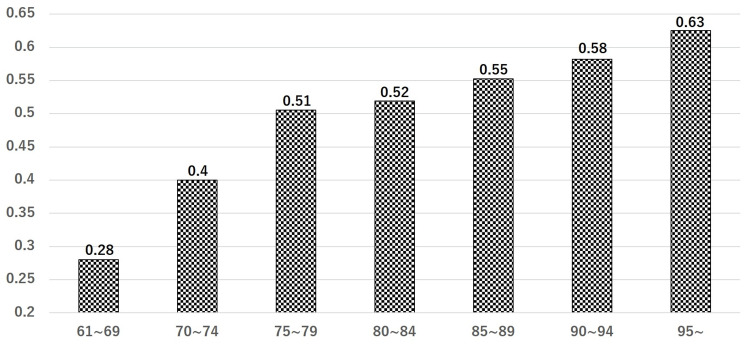
Ratio of A2 fractures A2 fractures increase with age in each category. A2: unstable

Of 808 patients, 371 (45.9%) were classified to have stable fractures (A1), and 437 (54.1%) had unstable fractures (A2). The mean and standard deviation at the age of surgery for patients in the A1 and A2 groups were 84.9±7.7 and 86.9±6.8, respectively. The number of patients for each age distribution of 61-69, 70-74, 75-79, 80-84, 85-89, 90-94, and 95 or older for the A1 and A2 groups was 18 and 7, 18 and 12, 43 and 44, 76 and 82, 107 and 132, 79 and 110, and 30 and 50, respectively, showing that the difference of categorial distribution was statistically significant (p=0.002) (Figure 2). To evaluate if the unstable (A2) fractures increase with age in each category, we calculated the ratio of A2, which is the number of A2 fractures divided by the total number of fractures per group. The values in each category of 61-69, 70-74, 75-79, 80-84, 85-89, 90-94, and 95 or older were 0.28, 0.4, 0.51, 0.52, 0.55, 0.58, and 0.63, respectively (Figure 3).

Overall, 278 females and 93 males were classified as stable (A1) fractures compared with 359 females and 78 males as unstable (A2) fractures (p=0.01). There were 166 right-sided and 205 left-sided stable (A1) fractures and 179 right-sided and 258 left-sided unstable (A2) fractures (not significant (NS)).

Timing of surgery

The total number of A1 and A2 surgeries by month is as follows: January with 69 (34 and 35, respectively), February with 62 (26 and 36, respectively), March with 57 (24 and 33, respectively), April with 64 (29 and 35, respectively), May with 51 (21 and 30, respectively), June with 37 (18 and 19, respectively), July with 63 (32 and 31, respectively), August with 50 (20 and 30, respectively), September with 67 (34 and 33, respectively), October with 62 (33 and 29, respectively), November with 64 (23 and 41, respectively), and December with 77 (37 and 40, respectively). December had the highest number of surgeries, and June had the lowest number of surgeries. There was no statistical difference in surgeries by month.

**Figure 4:**
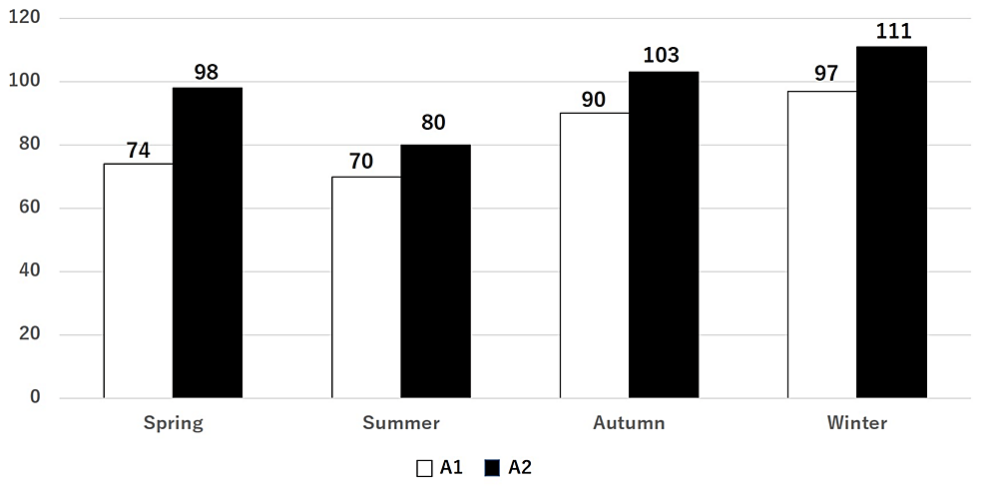
Number of A1 and A2 surgeries by season The seasonal frequency in descending order was winter, autumn, spring, and summer. The difference in fracture stability in each season was statistically insignificant. A1: stable, A2: unstable

The total number of A1 and A2 surgeries by season are as follows: spring with 172 (74 and 98, respectively), summer with 150 (70 and 80, respectively), autumn with 193 (90 and 103, respectively), and winter with 208 (97 and 111, respectively) (Figure 4). There was no statistical difference in surgeries by season.

## Discussion

Our study demonstrated that of the 808 patients, 371 were classified as stable (A1) fractures and 437 as unstable (A2) fractures, with higher reliability using 3D CT-based imaging than plain radiographic images alone. The demographics of these patients with regard to fracture classification, age, gender, fracture site laterality, and surgery timing (month and season) were surveyed. Our results showed that the total number of patients with pertrochanteric fractures (A1 and A2) increased with age, peaking at the 85-89 age group, and the number of unstable fractures (A2) is more prevalent in the older age groups (80 years and above), which is in accordance with other studies [14]. In this study, there were more female patients in the older age groups with A2 fractures compared to male patients.

The results of studies on pertrochanteric fracture using either the traditional or the revised AO/OTA classification varied depending on the imaging used, i.e., plain radiographs or CT scans. Several studies using plain radiographs and the traditional AO/OTA classification reported a 60% rate of unstable (A2) fractures [15,16]. A classification study in Norway also used plain radiographs [17]. In a separate study using CT imaging and the revised AO/OTA classification, 52.2% of fractures were classified as unstable (A2) [18], which is similar to our result of 54.1%. The difference in results among these studies may be related to two main factors. First, plain radiograph-based classification studies underestimate fracture stability due to the difficulty in assessing the posterior osseous fragments, hence leading to possible errors in classification [11,17,19]. In our preliminary study, 20%-40% of A1-classified fractures using plain radiographs alone were reclassified as A2 using 3D CT scan images. Second, the difference in the traditional and revised AO/OTA classification, e.g., a traditional A2.1 fracture will be classified as an A1.3 fracture in the revised system.

Strengths

The first strength of this study is the use of a homogenous cohort with a complete set of two-directional plain radiographs and 3D CT scan images leading to higher reliability in fracture classification. The second strength of our study is that fracture classification was conducted by a single surgeon with knowledge of the revised AO/OTA classification system, effectively preventing misclassification, as may be seen in studies using national registries or mail-in systems [13,19]. It is usually difficult to detect the knowledge of reviewing surgeons, as this would greatly affect the reliability of classification between pertrochanteric and subtrochanteric fractures [19,20]. In our current study, 72 of 989 patients from our surgical database were excluded as they were classified as presumed subtrochanteric fractures by three separate reviewers. Differences in fracture classification could therefore be enhanced in epidemiological studies that employ several anonymous observers across multiple centers. Another strength of our study is that our data were obtained from five different hospitals in separate locations, giving our data a good representation of the regional population.

Laterality of fractured site and fracture classification

In a study using the Swedish Fracture Register of 10,548 patients, there was an even distribution of right and left fractures [16]. Other articles reported that left-sided fractures occurred more frequently than right-sided fractures [14,18]. Similarly, our study had more left-sided than right-sided fractures (463 left and 345 right). There were 205 left-sided and 166 right-sided fractures classified as A1 and 258 left-sided and 179 right-sided fractures classified as A2. There was no statistical significance regarding laterality and fracture classification. To our knowledge, there are no reports on the relationship between fracture laterality and fracture stability.

Timing of surgery

Pertrochanteric fractures are more frequent during the winter months [16]. Studies conducted in Sweden, Spain, and Norway all showed higher incidences of fractures during the winter season [16,17]. Our data is in accordance with these reports with 208 total fractures during the winter (97 A1 and 111 A2). The factors reported to contribute to the higher rate of fractures during the winter months include slippery ground conditions, thick garments that hinder normal agility, inadequacy of home safety precautions, and a decrease in exposure to sunlight leading to decreased vitamin D synthesis essential for bone metabolism [21]. On the contrary, most fractures occur indoors or at home, and lack of vitamin D exposure during the winter months may be too short to decrease osseous strength.

Limitations

Our article has several limitations. The first limitation is the sample size of 808 patients, which is smaller compared to other demographic studies with a sample size of several thousand patients [13,14,16]. Nevertheless, regarding the use of CT images in AO/OTA classification studies, our sample size is larger than most studies, with a previously reported maximum of 203 [22]. The second limitation is related to the variables wherein only age, gender, and laterality were evaluated, not including the cause of the fracture nor other comorbidities such as osteoporosis or diabetes mellitus [23]. The third limitation is the time period of the study (January 2015 to December 2020), which may be epidemiologically inaccurate due to the possible influence of COVID-19. However, we believe that our current demographical survey is less likely influenced by COVID-19 despite the inclusion of the year 2020, as proved by surveying reports [24,25].

## Conclusions

In our demographic study of 808 patients over 60 years of age diagnosed with pertrochanteric fractures, 637 were females and 171 were males. Although using 3D CT-based images plays a more reliable role in the revised AO/OTA classification than plain radiographic evaluation, 371 were classified as stable fractures (A1) and 437 were unstable fractures (A2). Unstable fractures were significantly more in females with an age peak of 85-89 years. Pertrochanteric fractures occurred more frequently in the winter (December to February) and less frequently in the summer (June to August).
